# Improving Mental Health Referral Systems in Rural Australia: Co-Design Study With Health Professionals and Consumers

**DOI:** 10.2196/73460

**Published:** 2025-09-18

**Authors:** Kate Bartel, Asini Malinka Siriwardene, Paul Worley, Kim Pearce, Niranjan Bidargaddi, Bronwin Patrickson, Simon Moody, Sharon Wingard, Martin Jones, Brian McKenny, Darryl Cameron, Sharon Lawn, Amy E Mendham

**Affiliations:** 1 Riverland Academy of Clinical Excellence, Riverland Mallee Coorong Local Health Network, 10 Maddern Street, Berri, 5343, Australia, 61 08 8580 2400; 2 College of Medicine and Public Health, Rural and Remote Health, Flinders University, Adelaide, Australia; 3 College of Medicine and Public Health, Flinders Health & Medical Research Institute, Digital Health Research Lab, Flinders University, Adelaide, Australia; 4 School of Nursing and Midwifery, Edith Cowan University, Bunbury, Australia; 5 Department of Rural Health, University of South Australia, Whyalla, Australia; 6 Barossa Hills Fleurieu Local Health Network, Angaston, Australia; 7 Moorundi Aboriginal Community Controlled Health Service Inc, Murray Bridge, Australia; 8 Lived Experience Australia, Adelaide, Australia

**Keywords:** co-design, digital solution, focus group, referral, mental health, rural health care

## Abstract

**Background:**

In rural Australia, geographical isolation, limited resources, and complex health care navigation create significant barriers to mental health care access. Mental health care professionals and organizations often work in segregation, exacerbating existing barriers. Digital technology provides an opportunity to improve communication between providers and streamline workflows while supporting a diverse range of consumers.

**Objective:**

This co-design study aimed to identify rural community needs and explore digital solutions to enhance mental health service delivery pathways.

**Methods:**

Using a design-thinking methodology, we conducted focus groups and workshops with 17 participants (7 consumers and caregivers and 10 health care professionals) from a rural region to understand mental health service needs, systemic challenges, and design potential digital solutions. Thematic analysis followed a grounded theory approach, involving systematic coding and theme development through an iterative consensus process.

**Results:**

Access to mental health care emerged as the central theme. Rural community participants reported strong community connections but faced challenges, including limited technological innovation and substantial travel burdens. Health care professionals highlighted critical systemic pressures: underresourcing, overwhelmed clinicians with extensive waitlists, and complex referral processes. Both groups identified overlapping barriers in service limitations and system navigation. During the design phase, we developed personas capturing consumer and health care professional experiences and conceptualized an integrated digital solution comprising a health care professional dashboard and a consumer-facing app with caregiver access to enhance service coordination.

**Conclusions:**

The study demonstrated strong stakeholder support for implementing an integrated digital solution to enhance rural mental health service delivery. Further research is required to build upon the solution prior to testing, optimizing, and scaling.

## Introduction

Mental health burden has significant economic and social impacts [1], particularly in rural areas [2,3], which typically have fewer trained professionals, limited services, and less service innovations compared to metropolitan areas [3-6]. In Australia, 7 million rural residents face substantial barriers to mental health care access. These obstacles include fewer resources, limitations in technology, lack of knowledge of services, distance to services, and a deficiency in culturally sensitive practices [7], with complex and often ineffective treatment pathways [3].

General practitioners (GPs), as the primary contact for mental health support [8,9], face considerable challenges when connecting patients with appropriate mental health care, primarily due to time constraints in determining suitable referrals and limited availability of services in rural communities [10,11]. Adding to the complexity, service availability, fee structure, and communication methods vary between providers and change frequently, reflecting poor integration between services. This exacerbates the problem caused by inefficiencies and complexities in the health care system, decreasing the efficiency of care provision and inadvertently resulting in potentially less effective care [2].

The Australian government has provided the Initial Assessment and Referral Decision Support Tool (IAR-DST), a clinical decision support tool to assist with mental health care level determination. While IAR-DST determines care need, clinicians require additional support to rapidly identify suitable local providers and optimize referral processes [12]. Without such support, rural GPs may struggle to initiate the right referral on their first attempt where limited service availability already constrains their options. This may lead to rework, delayed treatment, and patient disengagement [13]. Moreover, many rural Australian people forgo seeking support altogether due to the difficulties in accessing GPs, so the importance of getting the referral processes right on the first attempt is even more imperative [10].

Digital referral platforms could augment existing assessment tools by automating provider matching and streamlining referral workflows [14]. Such systems show promise in reducing administrative load while supporting culturally and linguistically diverse consumers, for example, through better matching of services and more informed options [15]. However, their utility in rural Australian settings remains unexplored. Awareness of the needs and context of the community, what resources are available, and how their systems operate is invaluable [5,8]. Thus, to explore whether this could be a useful strategy, the community in which it would be implemented must be involved in the co-design process.

The primary aim of this formative study was to explore digital solutions for mental health care referral pathways in a rural community using participatory and human-centered design-thinking methods. Specifically, this study explored if the rural community wants a digital solution for mental health referral pathways, how it would be useful, and who would benefit.

## Methods

### Study Design

**Figure 1.**
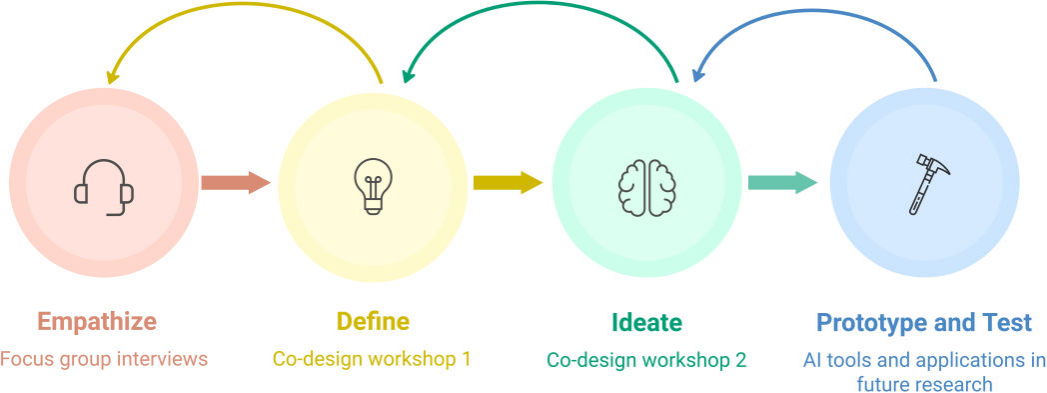
Participatory and human-centered design-thinking methodologies (adapted from NNGroup: Design thinking 101). AI: artificial intelligence.

Participatory and human-centered design-thinking methodologies informed the structure and approach for all participant activities (Figure 1). Design-thinking methodology is an iterative, user-centric process that aims to create a solution that is desirable, feasible, and viable [16] to end users, stakeholders, and the supporting infrastructure. Accordingly, consumers’ and health care professionals’ lived experiences, preferences, and needs are prioritized to develop a technological solution to improve referral efficiencies in rural communities.

### Design-Thinking Methodology

Design-thinking phases were reflected in the study design:

1. Empathize: Semistructured focus groups with consumers and health care professionals to develop a deep understanding of the rural community needs.
2. Define: A workshop to start the co-design process with combined consumer and health care professional input to prioritize the pain points in the mental health care system.
3. Ideate: A second workshop with consumers and health care professionals to brainstorm possible solutions.

### Setting

The study took place in the Riverland, South Australia, which is a small, rural region with a high level of rurality (Modified Monash Model 5), which entails considerable geographical remoteness and smaller population sizes. The Riverland area represents a typical low-resourced and low socioeconomic rural context with a Socio-Economic Indexes for Areas score of 996.8, indicating a level of socioeconomic conditions that falls below the national average.

### Participants

Consumers were recruited through researcher and clinician networks and included those with lived experience of mental health issues and formal or informal caregivers of people who experienced mental health issues. To be eligible to participate, consumers needed to be 18 years of age or older and have interacted with a health service regarding their mental health, or the mental health of a person they cared for, within the past 24 months. Health care professionals were recruited using purposeful sampling. Those who worked in the Riverland region in South Australia and had experience working with mental health consumers in the previous 24 months were invited to participate. The services approached included all public health sites, private mental health services, nongovernment organizations (NGO), charities, and Aboriginal services. Prospective participants were contacted via email or telephone and informed of the nature and purpose of the study, prior to consenting.

### Ethical Considerations

This project was approved by the Department of Health and Wellbeing Ethics Committee (2023/HRE00227) and the Aboriginal Health and Research Ethics Committee (04-23-1104). All participants provided written consent prior to their inclusion in the study and were remunerated for their time according to South Australian Health Department guidelines. Participants’ data were deidentified and stored on a secure electronic server.

### Data Collection

Data collection occurred from June to September 2024.

#### Empathize: Focus Groups

All focus groups followed a semistructured format as provided in Multimedia Appendix 1. Consumers (n=7‐10) participated in two 90-minute, face-to-face focus group discussions that were facilitated by experienced coinvestigators (KP and SL), including a lived experience representative (SL). The first session explored past experiences with mental health care and expectations of mental health management. The second focus group discussion explored current digital engagement behaviors and willingness to explore digital solutions. Health care professionals (n=7‐10) participated in two 60-minute focus group discussions conducted on the web (via Microsoft Teams) with the same facilitators. The first session explored the coordination of care and navigation across the mental health system. The second focus group discussion centered on experiences with technology as a solution and willingness to explore further a digital solution.

Following the completion of the focus group interviews, all participants were invited to participate in two 3-hour face-to-face design workshops. The workshop was facilitated by 2 external design-thinking experts. A consumer representative from a consumer and caregiver mental ill-health advocacy group (SL) and members of the research team with a background in psychology or focus group research assisted (KB, AEM, and AMS). All face-to-face focus groups and workshops were held in a local hospital meeting room.

#### Define: Design Workshop 1

Participants were asked to choose from consumer scenarios presented (as outlined in the Results section). For each scenario, consumers and health care professionals worked together in groups (n=4‐6) to map the key interactions that happen along the consumer mental health “journey.” Following the mapping process, these interactions and timelines were discussed with the entire group to fill in any gaps and highlight barriers in the system (pain points).

#### Ideate: Design Workshop 2

Key journey moments across the scenarios in workshop 1 were prioritized for conversation in workshop 2. Using these “key moments,” the room was split into 2 groups (1 group of consumers and 1 group of health care professionals). Each group discussed the pain points in the referral pathways and identified possible technological solutions. Following these small group discussions, the core pain points and technological solutions were discussed with the entire group. Following on from earlier conversations, participants were provided with a scope to focus on interactions between using mobile phone apps and health care professional–facing dashboards.

### Data Analysis

Focus groups were recorded, transcribed, and deidentified. Field notes were written by AMS and KB. Data were thematically coded using qualitative analysis software NVivo (version 14; Lumivero), guided by a grounded theory approach. The thematic coding process was managed by AMS and KB, in discussion with AEM, BP, and SL. Open coding was performed by AMS and checked for accuracy by KB, with any discrepancies discussed and resolved through consensus. Axial and selective coding were subsequently conducted by AEM, AMS, BP, KB, and SL [17]. Saturation of data was discussed (ie, no new themes were being generated by the data). Themes and subthemes were finalized by AEM, AMS, BP, KB, and SL. This study followed the COREQ (Consolidated Criteria for Reporting Qualitative Research) reporting guidelines, ensuring transparency in reporting researcher reflexivity, study design, and data analysis (Checklist 1) [18].

The iterative process of design-thinking methodology was implemented throughout the study, with data collected and analyzed in each phase to advise the next phases of data collection. Participants were provided with a verbal summary of previous session content, at the commencement of the session, and given the chance to comment or provide correction. Preliminary analyses of the focus groups were discussed with the research team, and external user-experience designers advised on the planning of co-design workshop 1. Participant outputs were digitally collated, and user personas were developed. User personas are a fictional representation of real-life users who share common characteristics, behaviors, goals, and pain points [19,20]. This effectively narrows down the intended users of the platforms and creates a more targeted approach, reflecting the “define” stage of the design-thinking process. Moreover, it creates a more detailed representation of the consumers to inform effective and targeted design decisions while maintaining the anonymity of the real study participants and reflecting their experiences accurately. Insights from co-design workshop 2 were similarly digitized and analyzed to identify key themes, which were then grouped to uncover opportunities. These opportunities were mapped back to the user personas to ensure that the solutions created serve the user.

## Results

### Overview

A total of 7 consumers and 10 health care professionals participated in focus groups and design workshops. In total, 10 consumers were contacted; 2 were unavailable, 1 did not attend, providing no notice, and 7 participated in at least 1 focus group or workshop. A total of 15 health professionals or organizations were contacted. In total, 3 professionals were unavailable, 2 did not return contact, and 10 attended a minimum of 1 focus group or workshop. Health care professionals included a psychiatrist, GPs (n=2), a clinical psychologist in private practice, NGOs and nonprofit managers (n=2), mental health nurses (n=2), and an Aboriginal nurse unit manager. Of the consumers, 5 had experienced a mental health issue, and 2 cared for someone with a mental health issue in the previous 24 months. One of the 2 Aboriginal consumers was an Aboriginal Elder.

**Figure 2.**
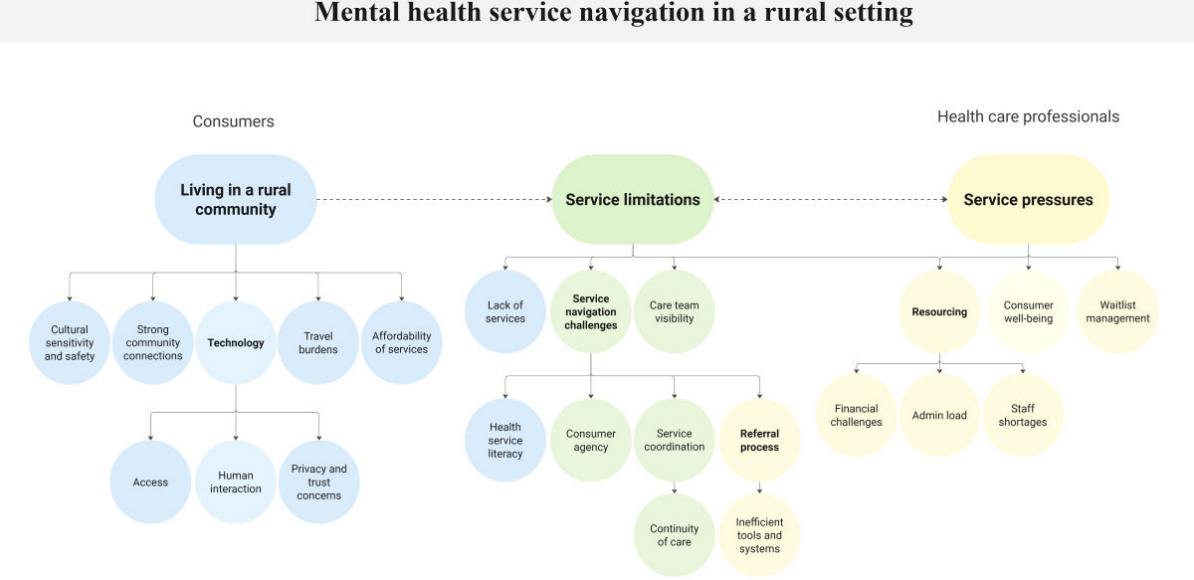
Coding tree of the main themes and subthemes identified from the focus group discussions.

The unifying theme identified across consumer and health care professional focus groups was “access to mental health care in a rural community.” A coding tree displays an overview of the main themes and subthemes identified from the focus group discussion (Figure 2), with example quotes provided in Table 1.

**Table 1.** Themes and subthemes from focus groups.

Themes and subthemes	Quotes
Theme 1: living in a rural community (consumer identified)	
1.1. Cultural sensitivity and safety	“I heard a whisper, they’re getting a morgue up here. I don’t know how true this is. But they want to know how to make it culturally appropriate. So they’re getting someone from Adelaide to come up here to tell us” [Consumer 2].
1.2. Strong community connections	“We are just one really, really big family” [Consumer 4—in relation to community group members in the Riverland].
1.3. Travel burdens	“Sometimes it’s transport because that’s terrible up here” [Consumer 1—assisting people with mental health support].
1.4. Affordability of services	“Clients don’t have money to pay to see a GP” [Health care professional 1].
1.5 Technology	
1.5.1. Access	“But I’m happy to use my phone. Get my SMSs when I’ve got appointments, they text me say your appointment” [Consumer 4]. “A lot of people who use our services don’t have smart phones. Or if they do, they have very limited capabilities and often don’t have access to internet” [Health care professional 2]. “I don’t know how to use a computer. But I know how to use my phone” [Consumer 4].
1.5.2. Human interaction	“I’d rather stand in a line at a grocery store rather than using the checkout because I like face-to-face contact” [Consumer 3—regarding worries about using technology]. “The least human interaction I can have sometimes, the better. If my social battery is full, empty actually, I’ll opt for that” [Consumer 5 regarding a fuel app that allows users to pay from their phone at the bowser].
1.5.3. Privacy and trust concerns	“Could this not just be—instead of, ‘How’s your Wednesday been? Let’s write this. What are your good thoughts, bad thoughts, gratitudes. Something to work on tomorrow.’ Instead of it being so invasive, could it not be a basic K10 every three weeks? Or just a check-in every so often to see how you’re travelling, instead of being an every single day thing” [Consumer 5].
Theme 2: service limitations (consumer and health care professional identified)	
2.1. Lack of services	“Up here, if you need to get referred onto any specialist service, like a psychiatrist or a psychologist. They’re just not available and you just can’t make an appointment with one” [Consumer 1]. “Specialist services not being available in Riverland” [Health care professional 6—in relation to the biggest pressure point they face in the Riverland with mental health services].
2.2. Care team visibility	“It would be helpful to know who is part of this client’s care team” [Health care professional 1].
2.3. Service navigation challenges	
2.3.1. Health service literacy	“I’m still not aware of all the services here or what’s available” [Consumer 3].
2.3.2. Consumer agency	“The person’s got to make a choice. It has to be their choice” [Consumer 1]. “Some sort of system that would involve the client, so that information that was being shared was accessible by the client and was maybe even directed by the client” [Health care professional 1].
2.3.3. Service coordination	“It would be really helpful in terms of me then, if I could somehow use that system to communicate with their GP or other people in their team” [Health care professional 5].
2.3.3.1. Continuity of care	“Not getting your own GP when you want to go and see the doctor” [Consumer 4].
2.3.4. Referral process	“When you look at how many services are in town, I don’t know what they all are. I don’t know who’s referred people to whom” [Consumer 1]. “Yes and if they require an actual official referral” [Consumer 5 in response to Consumer 1].
2.3.4.1. Inefficient tools and systems	“Depending on the GP, I find it varying levels of useful” [Health care professional 5—addressing referrals they receive from general practitioners]. “The other thing is that when the clinician who’s assessing the patient at the intake, if that assessment is just limited to a tool or is too superficial or is mostly looking at past records. Especially for risk assessment, then usually they get it wrong” [Health care professional 6].
Theme 3: service pressures (health care professional identified)	
3.1. Consumer well-being	“A lot of clients are completely disengaged with mental health services, despite them being significantly unwell” [Health care professional 2]. “I think it would be beneficial for lots of family and carers to be involved and have access to the apps, because they’re obviously usually the first to know when people are deteriorating. It gives them resources too, how to navigate the step model of care for their person” [Health care professional 1].
3.2. Waitlist management	“I find waitlist management really hard, because it’s impossible for me to predict how long someone’s going to need services for” [Health care professional 5].
3.3. Resourcing	“This hospital’s constantly bed blocked now. I work closely with [psychiatrist] and the on-call psychiatrist to determine whether community treatment would be more beneficial” [Health care professional 3].
3.3.1. Financial challenges	“Or finances which is obviously what [Health care professional 6] has sort of alluded to as well” [Health care professional 4—in response to the biggest mental health service challenges faced].
3.3.2. Admin load	“It’s another administrative job that nobody wants to do” [Health care professional 1].
3.3.3. Staff shortages	“Recruitment and retention of staff is a big one” [Health care professional 4—in response to the biggest mental health service challenges faced].

### Empathize: Focus Groups

#### Overview

Consumer insights were primarily shaped by the experience of living in a rural community. Such experiences were underpinned by the effects of geographical isolation and lower population densities, often resulting in service limitations. These limitations may, in turn, stem from broader service pressures faced by health care professionals such as staff shortages and a lack of resources, contributing to the unique challenges faced by rural communities in accessing mental health care.

#### Living in a Rural Community

In spite of the challenges faced, consumers living in rural settings had strong community connections, describing themselves as “one really, really big family” (Consumer 4), feeling a sense of pride in residing in the country. Aboriginal participants echoed these sentiments by having support networks to care for those with mental health issues. Regardless of strong community connections, trust in health care systems was varied, with Aboriginal participants less likely to trust in health care systems. As an Aboriginal Elder observed: “That’s where it’s different for Aboriginal people. The health system was where they stole kids” (Consumer 2).

Historically, Aboriginal people in Australia have faced systemic discrimination [21], leading to distrust in the health care system, underscoring the importance of cultural safety and sensitivity when developing the technological solution. Distrust in health care systems may also extend to distrust in technological products issued by these systems, as corroborated by consumers, regardless of Aboriginality. Lacking cultural and rural appropriateness, privacy, and data mishandling concerns may contribute to this varied trust.

When asked regarding the general experience of using digital technology tools, consumers raised concerns about accessibility, as some were unable to afford smartphones, phone credit, or had limited digital literacy.

Consumers reinforced that rural mental health services were harder to access due to affordability or geographic location, often requiring numerous visits to metropolitan Adelaide for specialist services. Of the services that were available in the Riverland, most were said to have long wait times, or consumers were not aware of their existence.

#### Service Limitations and Potential Solutions

Health care professionals and consumers voiced that available mental health services were hard to find and difficult to navigate for consumers in rural areas. Despite being a lifelong resident in the Riverland, a consumer was “still not aware of all the services here or what’s available” (Consumer 1), and another reinforced that it would be helpful to “create some kind of a roadmap to the services” (Consumer 5). This lack of health service literacy may be a by-product of ineffective communication and coordination between mental health services, GPs, and hospital services. Participants identified that effectively increasing interservice communication can lead to a better continuity of care, improving mental health outcomes for patients.

Care team visibility and effective communication may assist in avoiding duplication of services, as “there’s a lot of overservicing that happens in [the mental healthcare] space” (Health care professional 1), thus reducing workloads and service limitations. Consumers suggested that this may empower and encourage them to actively participate in their care while reducing the risk of confusion and misunderstanding. Consumers felt strongly regarding patient choice, having the necessary information to make an informed decision regarding their care. In this respect, the role of the caregiver was also emphasized by both consumers and health care professionals, as caregivers can advocate for patients when they cannot do so themselves. Caregivers are also “usually the first to know when [patients] are deteriorating” (Health care professional 1). Health care professionals respectively advocated for the consumers, valuing the importance of care “directed by the client” (Health care professional 1) and sharing information with the consumers.

#### Service Pressures and Potential Solutions

The predominant challenges and frustrations for health care professionals were categorized under the central theme of service pressures. Services were impacted by limited resource availability, which included a lack of finances for infrastructure and increased administrative loads due to staff shortages and attrition.

Most mental health services in the Riverland were characterized by prolonged waiting periods for patients, as providers were overburdened. Methods for the management of waitlists varied between health care providers. Participants suggested that if consumers were able to inform the health care service of their symptoms improving or worsening, health care professionals would be able to reprioritize consumers and better manage waitlists.

While both consumers and health care professionals agreed that displaying waitlists in the technology tool would be quite helpful, services would struggle to update waitlists regularly, as it would be the “first thing that slips when practices get busy” (Health care professional 1).

Health care professionals unanimously agreed that referral processes between services were underoptimized and “siloed” (Consumer 1), significantly impacting service delivery. Each service used varied processes and systems for referral and interservice communication, and most relied on inefficient and archaic tools such as fax machines: “It can actually delay consumer care because the fax might not be working, or got rejected” (Health care professional 3).

All health care professionals except 1 were unfamiliar with the IAR-DST, preferring practical, more familiar tools that allow flexibility in clinical judgment. Clinician discrepancies in tool use were often not visible to other health care professionals, impacting the efficiency of the referral process. In a similar vein, out-of-date waitlists and service directories place pressure on GPs to efficiently refer patients. For developing the technological solution, health care professionals prioritized consumer advocacy and recognized the importance of addressing consumer pain points in improving service provision.

### Define: Design Workshop 1

#### Overview

Workshop 1 was conducted with mixed groups of health care professionals and consumers. Based on the preliminary insights from focus groups in the empathize stage, the following consumer scenarios were developed.

1. An older person living alone in an isolated rural location with limited family support, moderate mental health challenges, and no internet access.
2. A young adult living and working in town with severe mental health challenges requiring frequent journeys between metro services and rural community support services.
3. A single parent with mild mental health challenges living rurally and accessing GP and NGO supports.
4. A young person attending a rural high school, accessing services and supports following an acute mental health admission at the local hospital.
5. A farmer living on a rural property, experiencing isolation and hesitant to seek support for his mental health challenges.

Participants chose scenarios 1, 2, and 3 for the journey mapping exercise. Each step of the consumer journey was mapped with the current channel that the consumer engages in, the value of this interaction, the software capabilities to replace or augment, the value that could be added from this augmentation, and the outcome for the consumer.

#### Scenario 1

An older person living alone in an isolated rural location with limited family support, moderate mental health challenges, and no internet access.

The role of the caregiver was particularly stressed in this scenario due to the consumer’s limited access to technology. All participants agreed that the caregivers should be given access to the technological solution at the discretion of the consumer. The caregiver will thus be able to facilitate consumer care through the digital tool.

Community support groups, or another trusted person, were proposed to facilitate mental health care support for consumers when a caregiver was unavailable. This was particularly pertinent for some Aboriginal consumers and those of a lower socioeconomic status, who may lack smartphones or internet access. Conversely, SMS text messaging through basic mobile phones appeared to be more readily available and accepted across the population. By incorporating an SMS text messaging feature into a clinician platform, accessibility may be enhanced for individuals with limited technological literacy or low socioeconomic backgrounds.

#### Scenario 2

A young adult living and working in town with severe mental health challenges requiring frequent journeys between metro services and rural community support services.

Current channels for patient-mental health service interactions were identified to be initial care visits (GP, hospital admissions, and community mental health groups), crisis services, specialized services, telehealth appointments, potential police contact, and ongoing support groups. Participants valued positive relational interactions in these channels, specifically feeling safe, respected, and avoiding behavioral stereotyping from decision makers such as law enforcement and doctors, as it can lead to inappropriate care.

Consumers and health care professionals reiterated that navigating the health care system with available services was overwhelming and confusing. Moreover, health care professionals acknowledged that patient handovers were inadequate, particularly between metropolitan and rural services. To address this, real-time information sharing, such as discharge summaries between services in the dashboard, was suggested.

Trust and privacy issues were identified as potential concerns for the clinician dashboard and consumer app. Both consumers and health care professionals emphasized the importance of data encryption to protect the transfer of sensitive patient data between services. Including the consumer in the care team ecosystem was suggested to entrust the consumer with handling of their own information, giving the ability to share or unshare data with selected providers.

#### Scenario 3

A single parent with mild mental health challenges living rurally and accessing GP and NGO supports.

Participants reaffirmed that navigating the available services in the Riverland was overwhelming, advocating for a centralized directory of services in the digital tool with customization specific to the user and their mental health requirements. The potential user in this scenario was presumed to have hectic schedules, leading to missed appointments or medications. To combat this, participants proposed the software to embed a medication and appointment management system that consolidates and integrates the user’s existing apps to prevent software fatigue.

#### User Personas

**Figure 3.**
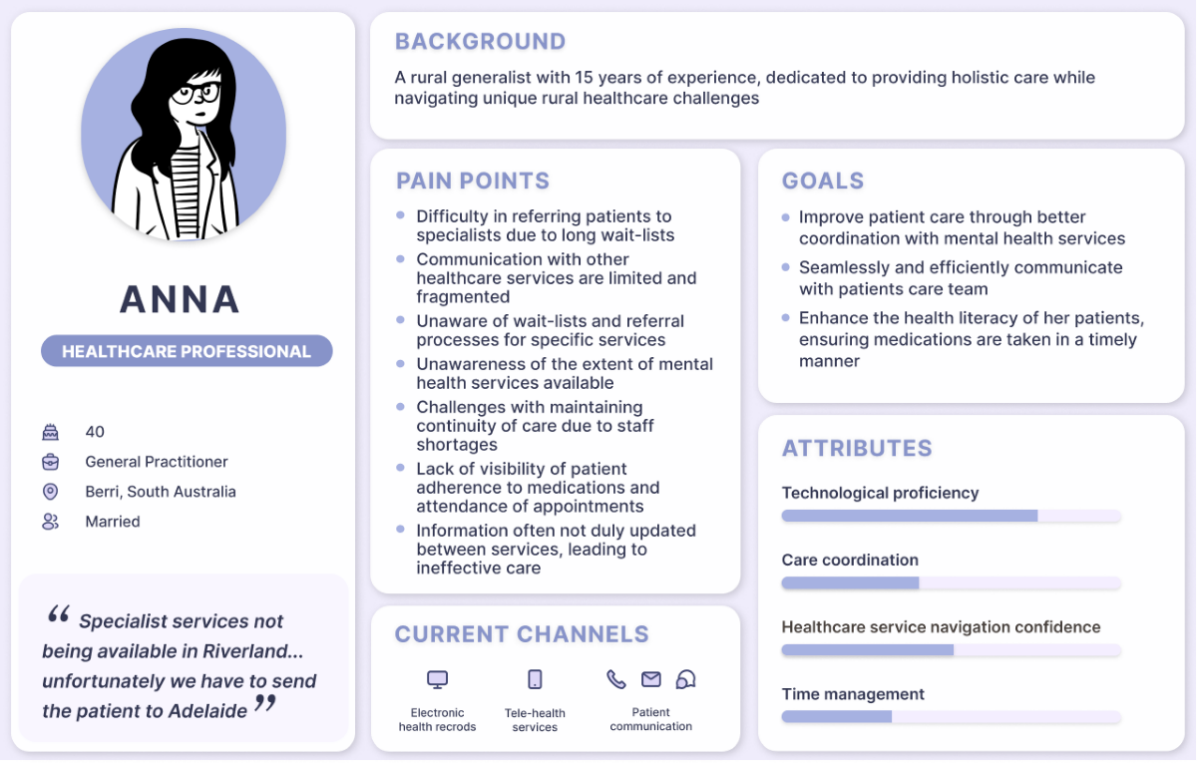
Health care professional persona “Anna”.

Informed by the insights derived from the 3 scenarios, the following user personas were developed (Figures3  4): “Anna,” a health care professional persona, and “Peter,” a consumer persona.

Health care professional persona “Anna” was structured around a GP, as this is often the initial point of contact for mental health care in Australia [22]. One of the biggest challenges in mental health care quoted by a clinician (Health care professional 6) was the lack of specialist services in the Riverland. Health care provider bottlenecks and frustrations presented previously are consolidated under pain points: inefficient referral processes, lack of communication between services, underresourcing, and difficulties in maintaining patient well-being. The desired outcome for Anna is to ultimately improve patient care by coordinating with other services effectively. The attribute scale displays Anna as proficient in technology, care coordination as poor, service navigation confidence as moderate, and time management as poor. Technological channels for providing care entail electronic health record systems and general practice software, telehealth services, and patient communication through mobile devices and email.

Consumer persona “Peter” was framed around scenario 2. Similar to health care professionals, access and availability of specialist services were quoted to be a pressure point by a consumer (Consumer 1). An amalgamation of consumer issues raised in focus groups and scenarios and pain points underscores rural access issues such as service access, long waitlists, and service navigation. Peter envisions more consumer-driven care and better support throughout his mental health care journey. The attribute scale presents technology proficiency as low, mental health awareness as moderate to high, health service navigation as poor, and access to technology as moderate. Peter’s current channels for accessing care include a GP for primary care, traveling for specialist services, and telehealth services for potential ongoing care.

**Figure 4.**
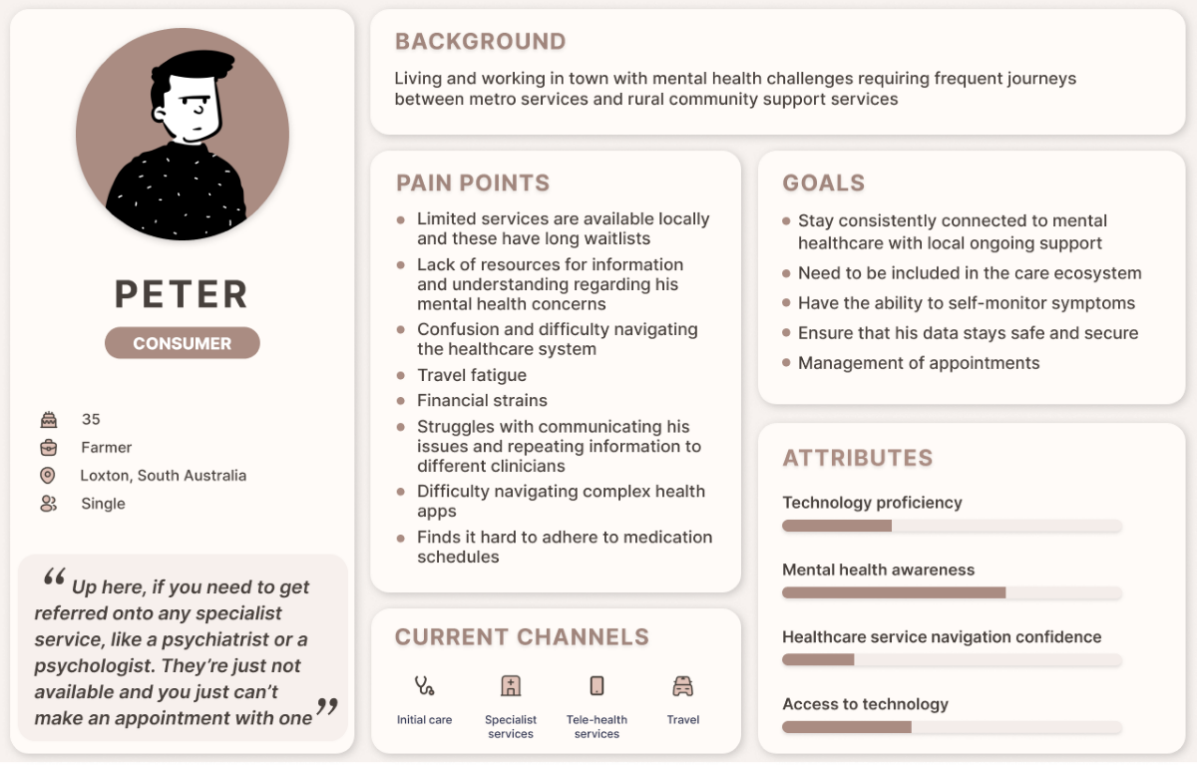
Consumer persona “Peter”.

### Ideate: Design Workshop 2

The content of the personas was used to guide participant discussions in workshop 2 and ensure that consumer and health care professional goals were kept central, and ideated solutions were addressing respective pain points.

**Figure 5.**
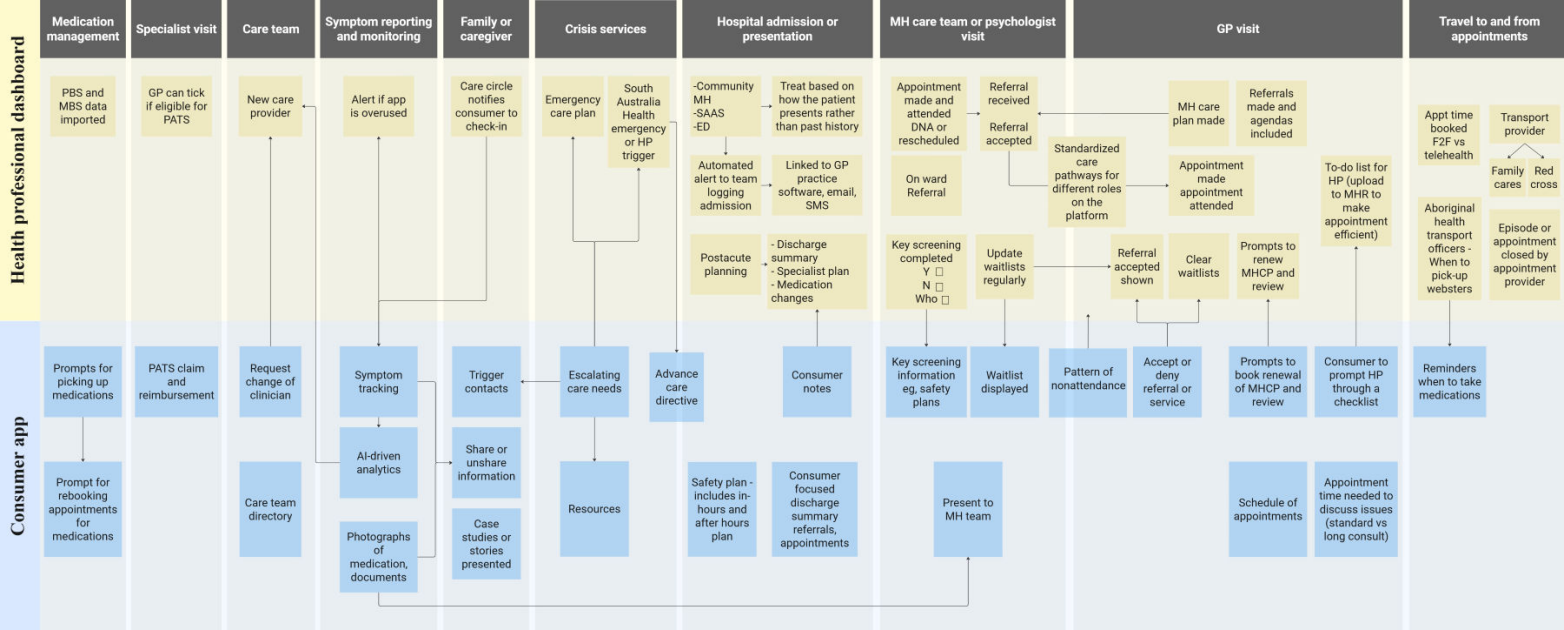
Interaction map outlining communication pathways within and between a health professional dashboard and consumer app. AI: artificial intelligence; DNA: did not attend; ED: emergency department; F2F: face to face; GP: general practitioner; HP: health care professional; MBS: Medicare benefits schedule; MH: mental health; MHCP: Mental Health Care Plan; MHR: My Heath Record; PATS: patient assistance transport scheme; PBS: pharmaceutical benefits scheme; SAAS: South Australia Ambulance Service.

Co-design workshop 2 iteratively revised bottlenecks in prior phases and generated possible solutions through an interaction map between the proposed clinician dashboard and consumer app (Figure 5). An overarching theme of “communication” was identified across the clinician and consumer journey. It was considered essential for health care professionals to be able to easily communicate with each other, and for the consumer’s journey to be easily visible, within the constraints of privacy and confidentiality. Health care professionals articulated the desire for consumer-specific information (eg, referrals made to other agencies) as well as generic organization information (eg, requirements for valid referrals), relevant to the consumer they are seeing, to be made clearly accessible. Likewise, communication was a key aspect of consumer agency, both for consumers being aware of their options and being able to communicate required information to their health provider, even if that was prompted via an app and discussed in a session. Participants also provided personal reflections of the study and its aim, ultimately affirming their support for a consumer-facing app and a health care professional–facing dashboard.

### Prototype and Test

Low-fidelity prototypes were developed by the research team based on ideated solutions and re-evaluated using the personas. These displayed key interactions between the consumer app and the health care professional dashboard. The main features will be applied and tested in the future include but are not limited to the connectivity between platforms used by different providers, self-assessment and patient assessment tools that are required for referral and monitoring processes, medication management, social network building, transport, and telehealth options.

## Discussion

### Principal Findings

The core purpose of this study was to initiate co-designing a digital platform with consumers and health care professionals, which may enhance referral processes and service navigation in rural mental health care. Three core themes emerged, which include (1) consumers identifying that “living in a rural community” has both positive aspects (strong community bonds) and negative challenges (access to technology and innovation); (2) health care professionals highlighting “service pressures” such as waitlists, consumer well-being, and resourcing; and finally, (3) both consumers and health care professionals identifying “service limitations,” such as navigation challenges and issues around an inefficient referral process. The human-centered technological solution that was explored in the workshops highlights the need for “communication” to integrate the consumer, caregiver, and providers, marking a critical step toward optimizing rural mental health care.

In this study, consumers highlighted both challenges and positive aspects of living in a rural community. Struggles included limited access to mental health services, often requiring travel to metropolitan locations for necessary care. Despite these challenges, rural consumers were often mentioned to be supported by dedicated caregivers and benefited from strong community bonds, especially among the Aboriginal population. These findings are consistent with prior studies [7] that identified barriers such as limited resources and distance to services while also highlighting the importance of person-centered and collaborative care. Thus, a key implication for future iterations of the prototype is to integrate community-based support systems to reduce strain caused by remoteness. Participants in this study further mentioned the importance of integrating other health services and community groups into the platform. While this is important for delivering a holistic service, it is outside the scope of this study and requires further investigation of limitations such as data sharing barriers. Future research could explore opportunities to integrate health services, providing additional support to consumers.

Rural health care professionals in this study faced pressures of underresourcing and access challenges. Similar to previous research [23], this resulted in mental health care systems that were overly complex, impacting health care professionals in coordinating care and consumers in navigating the required care. Our study showed that both providers and consumers deemed the current referral processes to be inefficient due to a lack of care coordination and limited communication between services, exacerbated by existing rural service limitations [7]. Participants in this study supported the view that a dashboard for health care professionals could address these referral inefficiencies by integrating referral tracking, communication tools, and standardizing or displaying referral processes for various services. Participants also agreed that a consumer-facing app complements the dashboard by offering consumers the agency to input information for their care team and displaying real-time service updates. An app could also allow for a consumer-nominated, trusted community member to access particular information, for example, appointment times. Thus, transport to appointments could be facilitated if the consumer is unable to take themselves. Although the digital platform may not directly resolve resource constraints, it is likely to reduce administrative loads for health care professionals, enhance care coordination, and provide a more consumer-centered approach to mental health care by improving the accessibility of services for consumers [24]. This could also afford a flow-on effect, whereby practitioner burnout is decreased and retention is improved. In particular, in rural locations where GPs take on a high proportion of mental health cases, easy, appropriate referral could reduce compassion fatigue and burnout [8].

Prior studies have explored the use of digital platforms to improve service navigation [15] and potentially improve communication among providers [25,26]. However, these platforms did not integrate well with existing infrastructure used by clinicians; thus, they resisted uptake [27]. Furthermore, many of these solutions were designed for urban settings and do not resolve the challenges faced by rural consumers and providers. Rural Australia, compared to metropolitan areas, has fewer public mental health systems [28] but a higher prevalence of private organizations that often operate in isolation. The health care professional dashboard could enable communication with these private organizations and public systems, facilitating care coordination. Thus, this study distinguished itself by its focus on a rural community. This uniquely positioned the study to co-design a platform to improve rural mental health referral pathways. Due to the location, there were fewer services to coordinate than a metropolitan region; yet, the population could benefit substantially from a platform that decreases the sometimes wastefully siloed service provision and enhances communication between service providers, consumers, and their care teams.

### Strengths and Limitations

A key strength of this study lies in its participatory approach, engaging both consumers and health care professionals in the design process, ensuring that the digital platforms developed are grounded in real-world experiences [29] and tailored to the unique challenges of rural mental health care. By using design-thinking methodology, the prototyped solutions are human-centric, contextually relevant, and functional [16]. The percentage of Aboriginal participants employed in this study was representative of the community demographics; however, further in-depth Aboriginal input is needed prior to implementing the solution. This study was also limited by its total number of participants. While this is reflective of a pilot study, it may also limit the generalizability of the findings to broader contexts; thus, future studies with a greater number of participants are necessary to further prototype the provider platform and consumer-facing app in continued consultation with end users. This includes testing the feasibility and acceptability and determining which cohorts would benefit most. Furthermore, the solution should be tested in other rural Australian regions to assess scalability.

### Conclusions

The proposed human-centered technological solution aims to integrate the consumer, the caregiver, and the provider, marking a critical step toward optimizing rural mental health care. The co-design process empowered consumers to actively engage in their mental health care journey while suggesting options to streamline system inefficiencies for overburdened clinicians. Ultimately, this platform may not only improve patient well-being and satisfaction but also boost the morale and retention of rural mental health professionals—a critical factor in addressing the current workforce shortage. Importantly, designing a solution in collaboration with rural communities may help to ensure a trusted, sustainable, and scalable implementation [29] in the future.

## Supplementary material

10.2196/73460 Multimedia Appendix 1 Focus group session guide.

10.2196/73460 Checklist 1 COREQ checklist.

## Abbreviations

COREQ: Consolidated Criteria for Reporting Qualitative Research
GP: general practitioner
IAR-DST: Initial Assessment and Referral Decision Support Tool
NGO: nongovernment organization
