# Supplementary material for: Improving Mental Health Referral Systems in Rural Australia: Co-Design Study With Health Professionals and Consumers
Source: JMIR Hum Factors. 2025 Sep 18;12:e73460. doi: 10.2196/73460 (PMC12445617; doi:10.2196/73460)
Supplement: Multimedia Appendix 1 [file humanfactors-v12-e73460-s001.docx]

Questions developed to guide focus group discussions by SL, KP, and KB.

**CONSUMER FOCUS GROUP 1**

***Aims***

- Identify challenges to accessing mental healthcare services
  - What is working, what isn’t

***Discussion points***

- Discuss adequacy and lived experience of accessing mental health services and support in the region.
- Expectations of mental health care service access
- What is your experience you while you wait for care/appointment availability? (before initial appointment/between appointments)
- Discuss consumer knowledge of existing services
- Discuss what it might look like to map your mental health journey
- Challenges to accessing mental healthcare services
- Possible solutions to accessing mental healthcare services

**CONSUMER FOCUS GROUP 2**

***Aims***

- Discuss barriers to engaging with technology and potential solutions
- Discuss enablers to engaging with technology

***Discussion points***

- Discuss: use of and experience with digital health tools
- How confident do you feel using mobile phone apps? What helps with mobile phone service usage for you?
- Barriers to engaging with technology and potential solutions and patterns of usage
- What information would be useful for you to know/obtain from an app (e.g., waitlist time, cost, provider location)?
- What information would you like to input into the app/what do you think it would be useful for a healthcare provider to know (e.g., wellbeing)?
- Privacy concerns and how you feel about carers accessing your data
- Bridge between reports that services keep and what consumer thinks about sharing of reports between health services

**HEALTHCARE PROFESSIONALS FOCUS GROUP 1**

***Aims***

- Discuss current services provided
- Current referral pathways and consumer monitoring
- Gaps in the region

***Discussion points***

- Discuss service demands and waitlist pressure points throughout the region.
- Has everyone heard of the Initial Assessment and Referral Decision Support Tool (IAR)?
- Are you currently utilising IAR and at what stage are you implementing this tool if so?
  - How do we compliment IAR?
  - How do we navigate the system that exists, for existing clients?
  - How do we monitor and address change in acuity level?
- Discuss cross-sectoral collaboration in mental health services.
- Identify current gaps in mental health services in your region.

**HEALTHCARE PROFESSIONALS FOCUS GROUP 2**

***Discussion points***

- Review pressure points identified in previous session.
- Discuss current interaction with technology.
- Identify opportunities to resolve those pressure points with digital health tools.
- Discuss potential digital referral pathways.
- What would you like to see in the future, regarding management of mental health referrals and changes in acuity?
- Identify opportunities to resolve pressure points with digital health tools
- Discuss potential risks and challenges of possible solutions
- What data is relevant to which people (e.g., wellbeing data)?
